# “Black bone”: the new backbone in CAD/CAM-assisted craniosynostosis surgery?

**DOI:** 10.1007/s00701-020-04445-z

**Published:** 2020-06-09

**Authors:** Bernd Lethaus, Dimitar Gruichev, Daniel Gräfe, Alexander K. Bartella, Sebastian Hahnel, Tsanko Yovev, Niels Christian Pausch, Matthias Krause

**Affiliations:** 1 Department of Oral and Maxillofacial Surgery, Leipzig University, Liebigstraße 12, 04103 Leipzig, Germany; 2 Department of Paediatric Radiology, Leipzig University, Liebigstraße 14, 04103 Leipzig, Germany; 3 Department of Prosthodontics and Materials Science, Leipzig University, Liebigstraße 12, 04103 Leipzig, Germany; 4 Department of Neurosurgery, Leipzig University, Liebigstraße 12, 04103 Leipzig, Germany

**Keywords:** Black bone, MRI, Craniosynostosis, CAD/CAM

## Abstract

**Background:**

Computer-assisted design and manufacturing (CAD/CAM) techniques have been implemented in craniosynostosis surgery to facilitate cranial remodeling. However, until now, computed tomography (CT) scans with ionizing radiation were necessary to plan the procedure and create guiding templates. The purpose of this study was to present our series using CAD/CAM techniques in planning and conducting fronto-orbital advancement surgery in patients with trigonocephaly with datasets acquired only by “black bone” magnetic resonance imaging (MRI).

**Methods:**

Six consecutively operated cases from 2019 were included in this study. All patients suffered from non-syndromic trigonocephaly with no primary surgeries. All patients underwent cranial MRI including black bone sequences. Preoperative planning and guides were created based on the DICOM datasets. We analyzed demographic data, clinical data, and outcome measured by Whitaker score.

**Results:**

In all cases, precise frontobasal advancement was possible with the CAD/CAM guides created by black bone MRI. The mean operation time and planning time were 222 and 32 min. The time on intensive and intermediate care unit (ICU/IMC) time was 4.5 days, respectively. All but one case were classified as Whitaker I.

**Conclusion:**

In trigonocephaly treatment by frontobasal advancement, black bone MRI-based CAD/CAM craniosynostosis surgery is safe and feasible. It offers the major advantage of completely avoiding CT scans and ionizing radiation with superior imaging quality of intracranial structures. Thus, it improves intraoperative safety and—at the same time—has the potential to reduce operating room (OR) time.

## Introduction

The aim of cranial vault remodeling is to create sufficient cranial vault volume, to allow better midfacial development, and to create an esthetically acceptable form. The use of computer-assisted design (CAD) and manufacturing (CAM) in the surgery of craniosynostosis has been described since 1996 [22]. Since then, many groups have implemented and refined this technique in their therapeutic procedures. Most surgeons use an aged-matched database that serves as a master model to create a normal cranial shape. Burge et al. formed different age-related bandeau templates that can be sterilized [1]. Others used averaged datasets of healthy children of different ages to create the ideal skull model [10, 14, 18, 21]. The advantages and benefits of these virtual and CAD/CAM-driven surgeries have been well documented. However, acquisition of three-dimensional (3D) datasets for the creation of models required computed tomography (CT) scans prior to surgery. Although the benefits of CAD/CAM procedures are without doubt, this adds another drawback due to the additional radiation exposure for very small children. The potential risks of ionizing radiation are well documented and are even more critical in young children [9, 11, 15].

Magnetic resonance imaging (MRI) has a broad application in cranial surgery, but the superior imaging quality of cortical bone on CT and the subsequent ability to create 3D reconstructions has largely outrun MRI for this purpose [4]. A new technique of MRI “black bone” has been reported to solve this problem by minimizing soft-tissue contrast and any signal returned from bone. The technique makes it possible to segment bone from the surrounding soft tissue to produce 3D-reconstructed images [4–6]. Recently, it has been shown that these datasets can be used to construct models using 3D printing [7].

The purpose of this study was to present our series using CAD/CAM techniques in planning and conducting fronto-orbital advancement surgery in patients with trigonocephaly with datasets acquired only by black bone MRI.

## Material and methods

### Study design

A retrospective review was conducted for patients treated in the craniofacial center of University Hospital Leipzig, Germany, with a CAD/CAM planned procedure for various indications for cranial vault remodeling in 2019. Patients were included with non-syndromic monostructural craniosynostosis of the metopic suture and resulting severe trigonocephaly. Exclusion criteria were syndromic craniosynostosis, signs of elevated intracranial pressure or hydrocephalus, history of previous cranial or intracranial surgeries, hemorrhagic diathesis, and severe comorbidities with potential impact on the perioperative course. A follow-up of at least 3 months was mandatory. The basic demographic and surgery-related data [presurgical planning time, length of hospital stay, length of intensive/intermediate care unit (ICU) stay, operative time, need for blood transfusion, intra- and postoperative complications] were documented. All data was extracted from the hospital’s patient organizing system (SAP, Walldorf, Germany), and statistical analyses were performed using IBM SPSS version 24. Due to the small amount of patients, only descriptive statistics were applied.

The postoperative outcome was assessed by assigning a Whitaker category based on follow-up descriptions by the neurosurgical and oral-maxillofacial team [23].

### Presurgical virtual planning

**Fig. 1:**
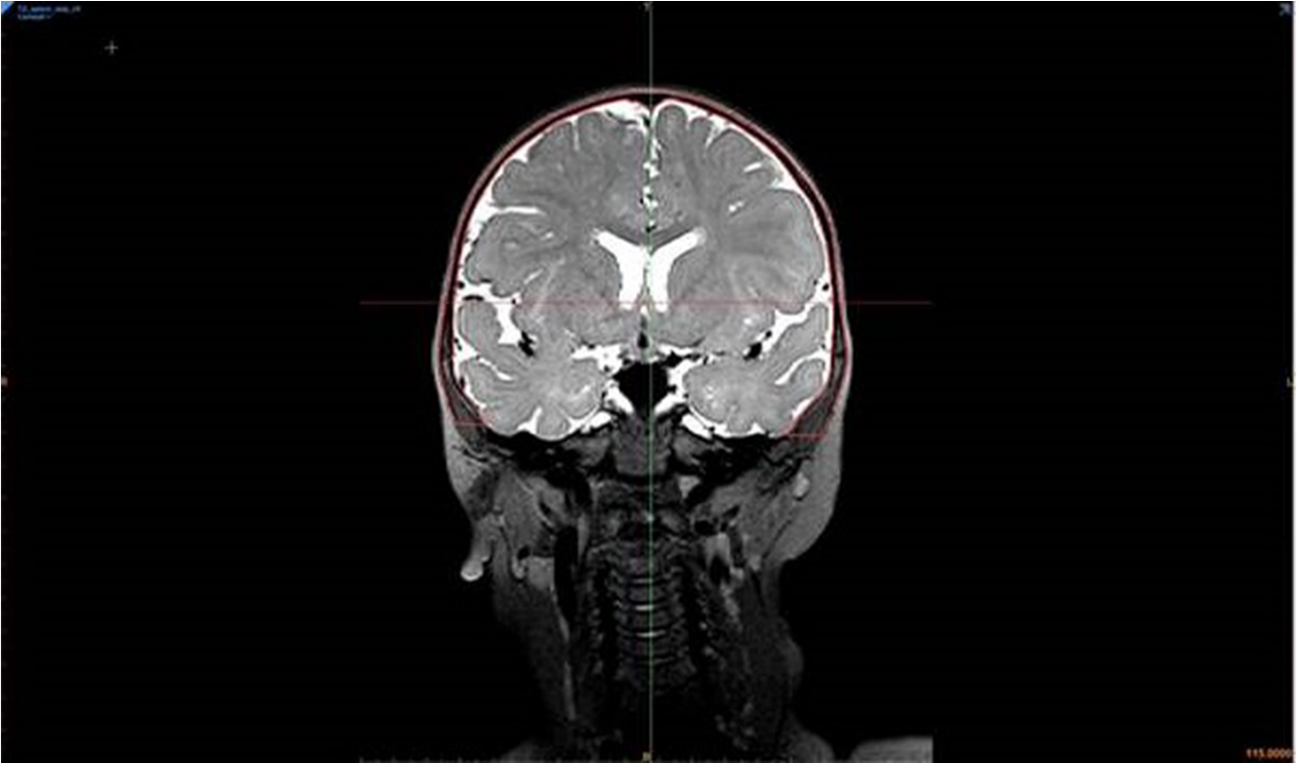
Screenshot of a MRI in coronal plane in a 1-year-old girl with trigonocephaly. The skull bone is segmented and traced with red lines

**Fig. 2:**
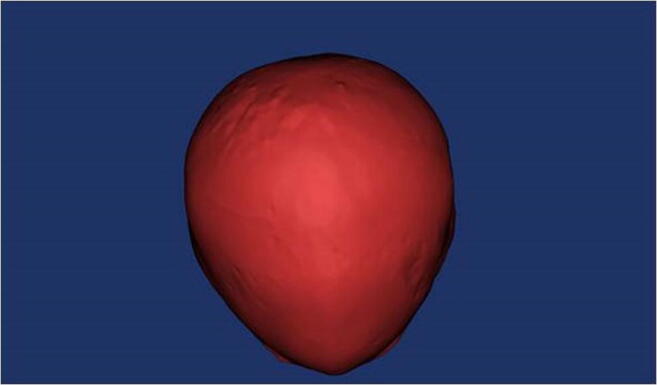
Extrapolated skull from black bone MRI dataset captured and segmented as seen in Fig. 1

All patients received a MRI scan under sedation prior to surgery. All examinations were performed on a 3T-MRI (Siemens Prisma Fit, Siemens, Erlangen, Germany) with a 64 channel array head coil. For the black bone sequence, the following parameters were employed: 3D gradient echo sequence, FOV 250 × 203 mm, voxel size 0.78 × 0.78 × 1.41 mm (interpolation to 0.78 × 0.78 × 1.0 mm), TR 1210 ms, TE 3.2 ms, flip angle 2°, and parallel acquisition technique GRAPPA 2. The average acquisition time for the black bone sequence was 3.5 min (Fig. 1). Osseous segmentation was performed (Fig. 2).

**Fig. 3:**
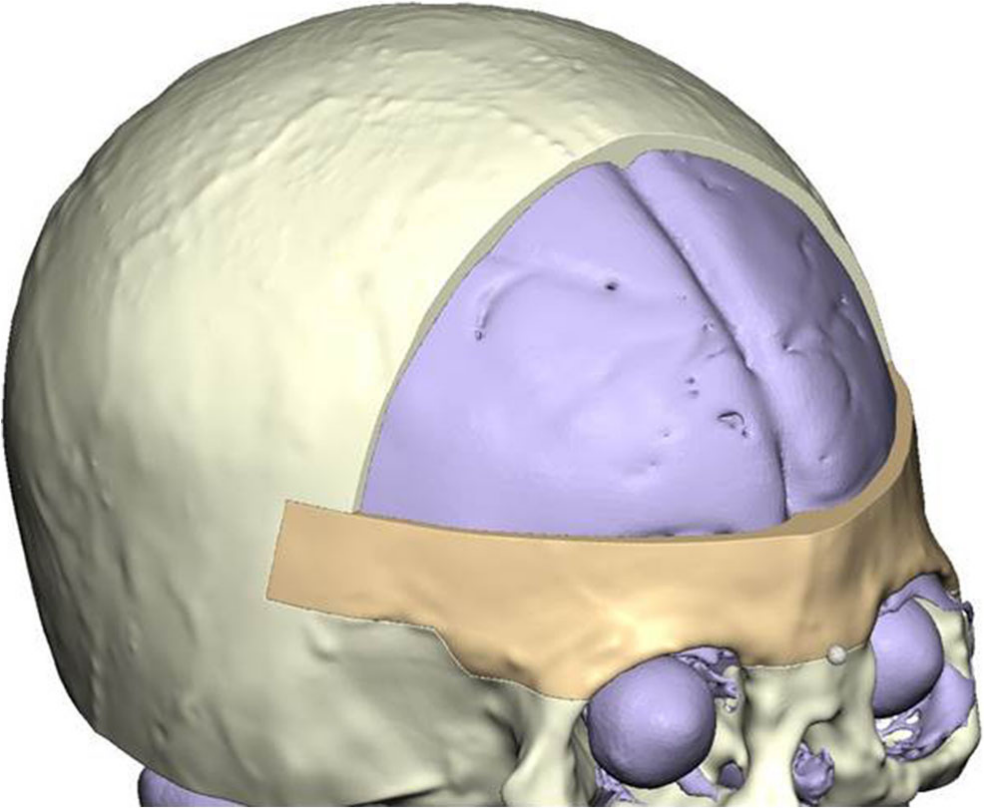
Virtual segmentation of the skull based on the black bone dataset. Emphasis was put on the frontal bone, dural surface, and position of arachnoid granulations. The course of the sinus is clearly visible between the hemispheres

**Fig. 4:**
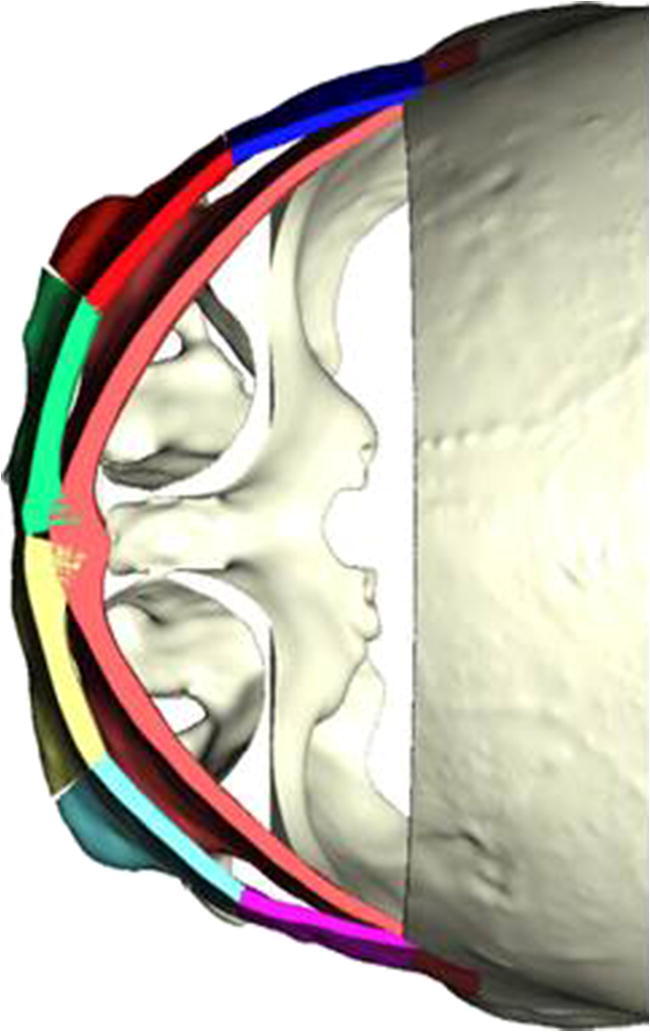
Form and placement of the bandeau before osteotomy (light red) and after segmentation and repositioning (multi-color). The upper frontal osteotomy has been virtually removed. Templates are printed according to the planning

The DICOM dataset was uploaded to a virtual platform for CAD/CAM-supported surgeries (IPS Gate KLS Martin, Tuttlingen, Germany). A specialized case designer created a 3D model from the DICOM data focusing on the soft tissue and bony segmentation of the skull. Further emphasis was put on the position of the brain sinus venous system (Fig. 3). The surgical procedure and osteotomies were planned with the surgeon and the technical specialist during a virtual meeting (Fig. 4). The osteotomies were positioned in the 3D models and controlled in the axial, sagittal, and coronal planes. Special attention was given to neurovascular anomalies and bone thickness. An age-dependent reference skull model was implemented in the planning to facilitate the virtual contouring and adapt the planned osteotomies. Based on the virtual plan, cutting guides and positive and negative molds of the planned operative results were produced and sterilized.

### Surgical procedures

**Fig. 5:**
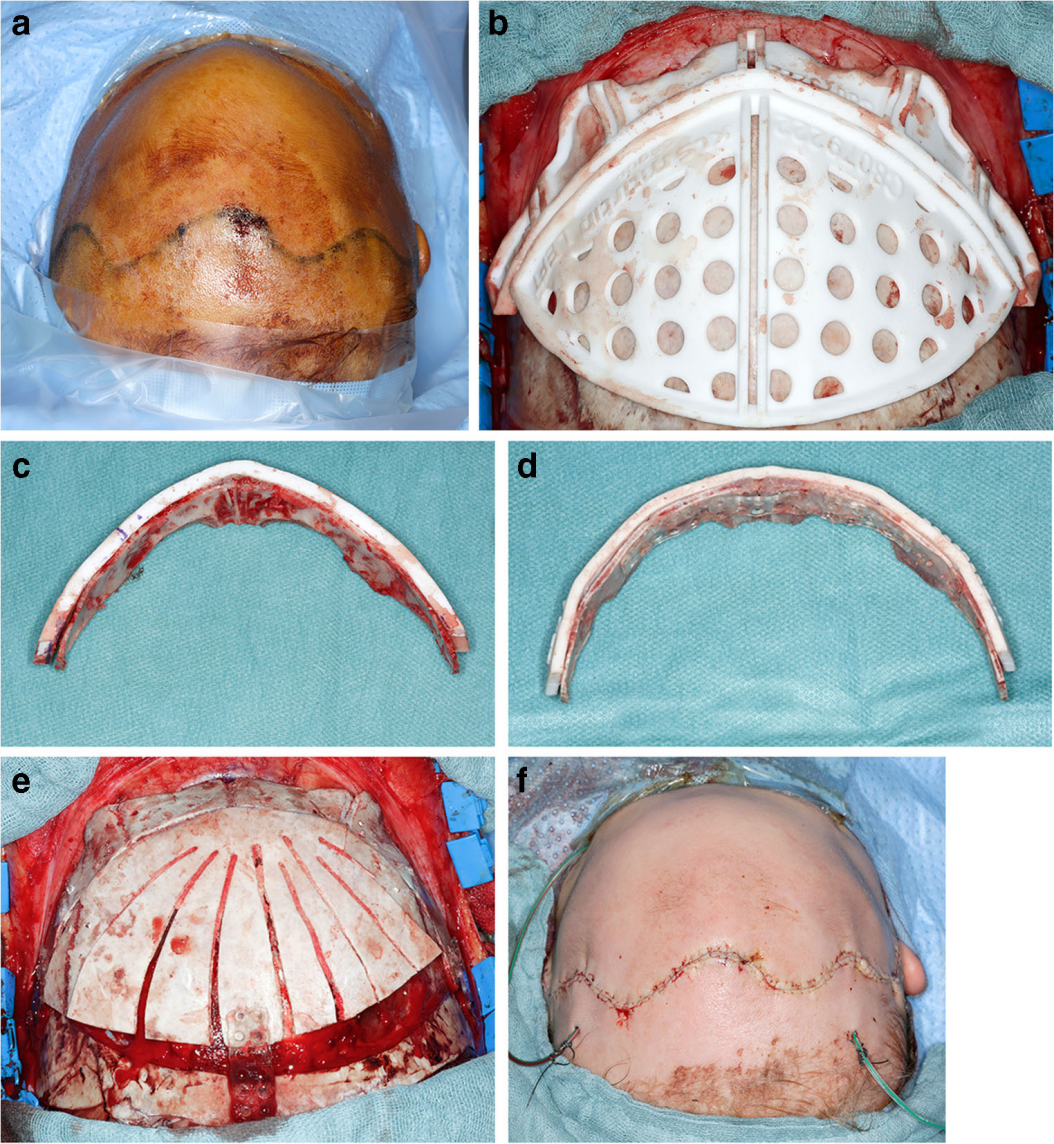
**a** Preoperative skull form of the patient in supine positioning. **b** Intraoperative placement of the cutting guides of the bandeau and frontal segments with precise positioning. **c** The bandeau was removed using the template and piezosurgery, and precision of the cutting guide is verified. **d** The bandeau was then adapted to the mold with the planned form and fixated with resorbable plates (inferior picture). **e** Form and position were fixed with the same procedure as for the bandeau with resorbable plates. The segments and bandeau were repositioned and fixed with resorbable plates to the outer aspect of the remaining bone. **f** Postoperative skull form of the patient in supine positioning

The use of cutting guides and molding plates followed the systematics described recently by others using CT-based datasets [2, 12, 20]. With the patient in the supine position (Fig. 5a), a wave-shaped incision was made ear to ear, and skull and facial areas were exposed. The cutting guides were placed on the bone and outlined with a pen (Fig. 5b). After removal of the cutting guides, the craniotomies of the frontal or occipital regions were performed. The fronto-orbital bandeau was removed using piezosurgery. In the bandeau segment, corticotomies were applied as indicated by the cutting guide. It was then adapted to the mold with the planned form (Fig. 5c, d). Resorbable plates and screws were used for fixation on the outer aspect of the bone segments for the cranial vault, and a few were used on the inner aspect of the bandeau (Sonic weld, KLS Martin, Germany). The calvarial segments were placed in the negative mold according to the planned position. Calvarial reshaping was performed by fan-shaped osteotomies. Form and position were fixed with the same procedure as for the bandeau with resorbable plates. The segments and bandeau were repositioned and fixed with sufficient resorbable plates on the outer aspect on the remaining bone (Fig. 5e). After placing a subgaleal drainage and wound closure, the patient was transferred to the pediatric ICU/IMC (Fig. 5f).

## Results

In 2019, six CAD/CAM planned patients with non-syndromic trigonocephaly were treated in our institution by fronto-orbital advancement. The mean and median patient age at surgery were 17.8/14.0 months, respectively (range 12–30 month; Table 1). All patients received a black bone MRI sequence within 6 weeks prior to the operation. All planning was done in a single session with one or both surgeons and one technician. The mean planning time was 55 min (range 24–78 min.) with a clear learning curve visible (Table 1). The process from planning to delivery took 3 to 4 working days.

**Table 1:** Results of six patients with trigonocephaly followed by fronto-orbital advancement

	Average	Range
Mean age of patients	17.8 months	12–30 months
Median age of patients	14.0 months	12–30 months
Planning time (preoperatively)	55 min	24–78 min
Operative time	214 min	138–285 min
ICU/IMC stay	4.5 days	3–6 days
Hospital stay	8 days	7–13 days

In all cases, the cutting guides were placed according to the planning. The accuracy of the 3D-printed guides and models were excellent. No corrections in positioning or fitting were necessary. All patients were operated upon by the same team of two experienced surgeons (BL and MK). The mean operative time was 214 min (range 138–285 min). There were neither surgical complications nor cerebrospinal fluid (CSF) leaks. The mean ICU/IMC stay was 4.5 days (range 3–6 d). The mean total hospital stay was 8 days (range 7–13 days) (Table 1). Neither postoperative complications nor infections were recorded.

All but one patient were classified by the team of neurosurgeons and maxillofacial surgeons with Whitaker class I.

## Discussion

CAD/CAM planning and surgery found its way into cranial vault remodeling many years ago [22]. The techniques used have been refined and adapted since. This development has been also supported by the use of these techniques in cranio-maxillofacial surgery when facial bone had to be reconstructed with microvascular bony transplants. However, some centers have declined this technique in cranial vault remodeling because all virtual planning has required DICOM datasets acquired by CT scans. It has been shown that under regular conditions, CT scans are not mandatory to acquire diagnostic information to perform craniosynostosis surgery [8, 19]. There is a broad consensus that ionizing radiation should be kept to a minimum, especially in small children [13, 16, 17]. In comparison with normal-sized adults, the image contrast-to-noise ratio (CNR) for neonates is a factor of four higher if the same kV and mAs are used, which demands special protocols to be used to reduce the radiation [15]. Conversely, one can argue that craniosynostosis repair in small children remains an extensive surgery with associated risks that are much greater than the risks associated with ionizing radiation [12].

**Fig. 6:**
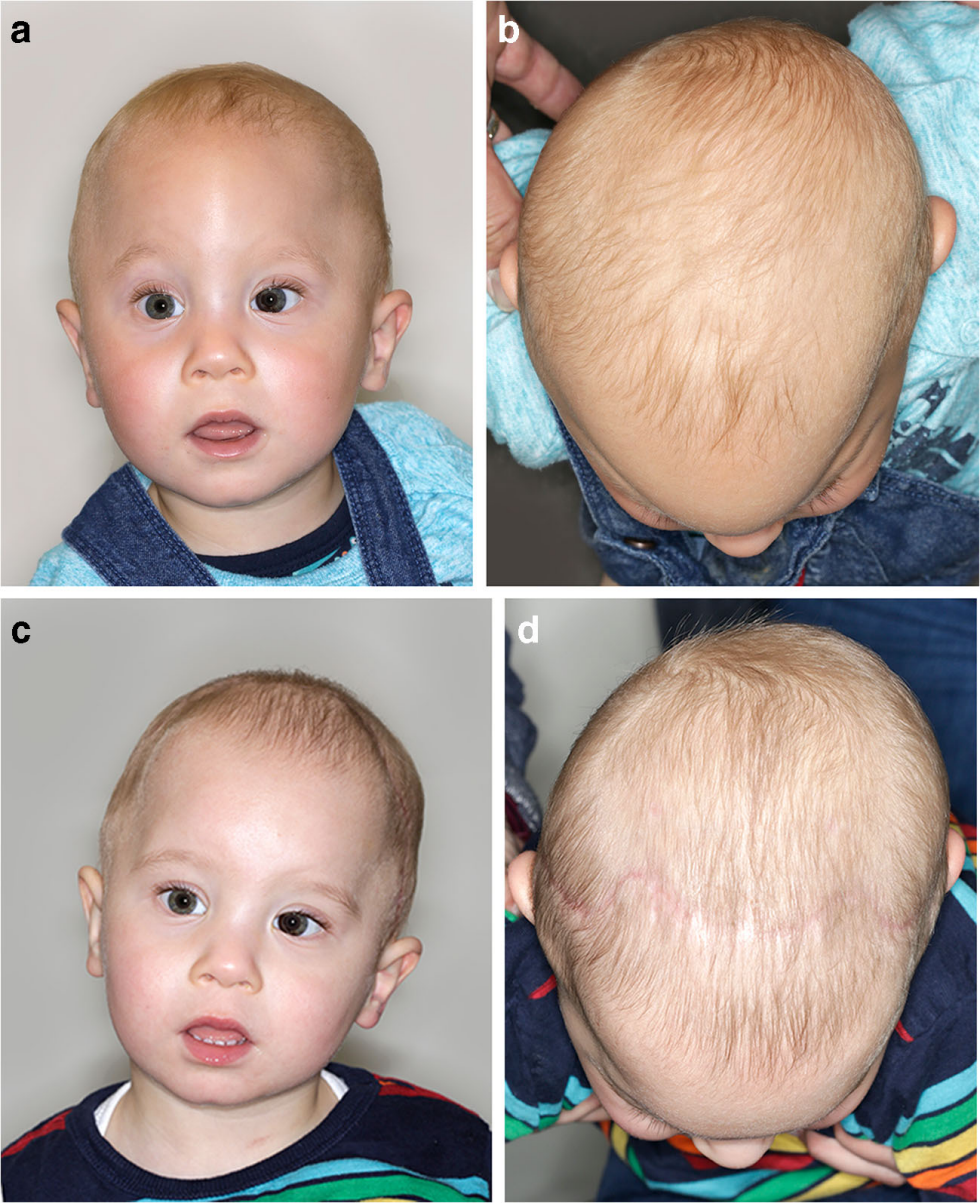
**a** En face preoperative image of an 11-month-old boy with non-syndromic trigonocephaly. **b** Image from a cranial perspective showing the triangle-shaped supraorbital segment and a mild posterior postural plagiocephaly. **c** En face image 3 months postoperatively. **d** Image from a cranial perspective after 3 months showing a harmonically shaped supraorbital segment

In this study, we were able to show in a small cohort the feasibility of CAD/CAM planning and producing cutting guides on the basis of “black bone” MRI in classic craniosynostosis surgery (Fig. 6a–d). Eley and coworkers have promoted this technique within in the last few years for cranio-facial indications. They recently described the 3D printing of a mandible due to “black bone” imaging [6].

To the best of our knowledge, this study is the first that implements this technique in clinical routine in craniosynostosis surgery. With MRI black bone sequencing, it is possible to achieve the sufficient precision of CT scanning to replicate skull and face surfaces that can be used for 3D printing to establish cutting guides [7]. All of our cases but one were classified as Whitaker I.

Superior illustration of the neurovascular and dural venous system, brain anomalies, and malformations is a further major advantage of MRI diagnostics. The additional information can be implemented in the planning and easily transferred to the cutting guides. Iyer et al. have shown that the knowledge of the precise location of these structures is variable, especially in asymmetric cases and can be integrated into the CAD/CAM planning. However, due to the superior soft-tissue resolution, MRI can facilitate this process. As always, in preoperative CAD/CAM planning, additional time has to be invested in the planning itself. In our series, planning required between 25 and 60 min, depending on the complexity of the case, and we observed a steep learning curve in the planning process. The same effect was observed in operating room (OR) time between first and last cases. There is still a need for a preoperative planning session between a technician and surgeon. The use of normative pediatric skull references facilitates the process [18], but the goal must be an automatic segmentation and sculpturing process such as an algorithm that is able to plan standard cases [21]. This would reduce planning time, the need of a technician, and presumably costs. Although we were not able to demonstrate this statistically, there is a high potential to reduce OR time for very young patients by meticulous preoperative planning as shown by other CT-based studies [3].

The small patient number and retrospective nature are limitations of this study. Further studies with larger numbers of patients and different indications will be necessary to evaluate this new method. Trigonocephaly as an indication has been a perfect start to test the implementation of black bone MRI in CAD/CAM planning. The bandeau with its irregular shape, small angles, and rapid change of convex and concave shapes secures a good fitting of the guides and reduces possible misplacements. This MRI-based system will become more challenging with indications addressing the posterior vault, requiring a flatter geometry. It is yet to be evaluated whether black bone MRI will be sufficient to produce guides with an accuracy high enough to allow only one possible positioning on the curvature of the skull.

## Conclusion

In trigonocephaly treatment by frontobasal advancement, black bone MRI-based CAD/CAM craniosynostosis surgery is safe and feasible. It offers the major advantage of completely avoiding CT scans and ionizing radiation with superior imaging quality of the intracranial structures. Thus, it improves intraoperative safety and has the potential to reduce OR time.
